# Nitric Oxide (NO) in Plant Heat Stress Tolerance: Current Knowledge and Perspectives

**DOI:** 10.3389/fpls.2017.01582

**Published:** 2017-09-13

**Authors:** Santisree Parankusam, Srivani S. Adimulam, Pooja Bhatnagar-Mathur, Kiran K. Sharma

**Affiliations:** International Crops Research Institute for the Semi-Arid Tropics Patancheru, India

**Keywords:** nitric oxide, heat stress, sodium nitroprussside, photosynthesis, antioxidants, lipid peroxidation, climate change

## Abstract

High temperature is one of the biggest abiotic stress challenges for agriculture. While, Nitric oxide (NO) is gaining increasing attention from plant science community due to its involvement in resistance to various plant stress conditions, its implications on heat stress tolerance is still unclear. Several lines of evidence indicate NO as a key signaling molecule in mediating various plant responses such as photosynthesis, oxidative defense, osmolyte accumulation, gene expression, and protein modifications under heat stress. Furthermore, the interactions of NO with other signaling molecules and phytohormones to attain heat tolerance have also been building up in recent years. Nevertheless, deep insights into the functional intermediaries or signal transduction components associated with NO-mediated heat stress signaling are imperative to uncover their involvement in plant hormone induced feed-back regulations, ROS/NO balance, and stress induced gene transcription. Although, progress is underway, much work remains to define the functional relevance of this molecule in plant heat tolerance. This review provides an overview on current status and discuss knowledge gaps in exploiting NO, thereby enhancing our understanding of the role of NO in plant heat tolerance.

## Introduction

An increase in temperature above the plant's optimum growth temperature that causes an irreversible damage to the growth is defined as heat stress (Wahid et al., 2007). Plants often encounter high temperature stress which disrupts plant metabolism and cellular homeostasis. More so in the coming decades, since ensuing steady increase in global temperature is predicted to have greater impact on biological processes (IPCC Climate Change, 2007; Johkan et al., 2011). Several reports indicate adverse effects of high and low temperatures on the molecular, biochemical, and physiological characteristics of plants (Suzuki and Mittler, 2006; Wahid, 2007; Mathur et al., 2011; Khan et al., 2013a; Chinthapalli et al., 2014; Brestic et al., 2016). Heat stress can cause oxidative burst, peroxidation of membrane lipids, pigment bleaching, protein degradation followed by enzyme inactivation and macromolecule damage in plants (Pospíšil et al., 2003; Suzuki and Mittler, 2006; Awasthi et al., 2016; Prasad et al., 2016; Santisree et al., 2017b). Heat stress has also been shown to disturb coordination of plant organelles, damage to the cytoskeleton, thereby altering cell differentiation and elongation (Smertenko et al., 1997). At times, high temperature lead to activation of silenced gene clusters nearer to the centromeric regions by inducing temporary loss of epigenetic gene silencing as shown in case of *Arabidopsis thaliana* (Lang-Mladek et al., 2010).

Plant responses to heat stress depend on plant developmental stage and strength of the stress condition (Brestic et al., 2012; Yamori et al., 2014). For example, in *Cicer arietinum*, exposure to high temperatures during the reproductive stage is more detrimental to yield when compared to the vegetative stage. Higher temperatures during anthesis lead to greater number of aborted and malformed buds, while at vegetative stage heat stress can negatively impact the development, morphology and nutrient uptake of the plant (Devasirvatham et al., 2012). Nevertheless, the optimum temperature varies among different compartments within the cell and species with in the genus (Pospíšil and Tyystjarvi, 1999; Hasanuzzaman et al., 2013; Chinthapalli et al., 2014). At times heat stress might lead to replacement of sensitive species by more heat tolerant species (Devasirvatham et al., 2012). A more recent research on 12 cultivars of *Oryza sativa* revealed a strong negative and differential impact of high night temperature on plant metabolism (Sharma et al., 2017). Hence, exploring the molecules that have the potential to protect plants from harmful effects of climate adversaries is gaining increasing interest among plant researchers (Hossain and Fujita, 2013; Mostofa and Fujita, 2013; Nahar et al., 2014; Wang et al., 2014; Savvides et al., 2016). Exogenous application of osmoprotectants, phytohormones, signaling molecules, trace elements have shown beneficial effects on plants under high temperatures, mostly due to their growth promoting and antioxidant capabilities (Uchida et al., 2002; Song et al., 2006; Khan et al., 2013b; Sagor et al., 2013; Mostofa et al., 2014; Nahar et al., 2014; Chan and Shi, 2015).

**Figure 1:**
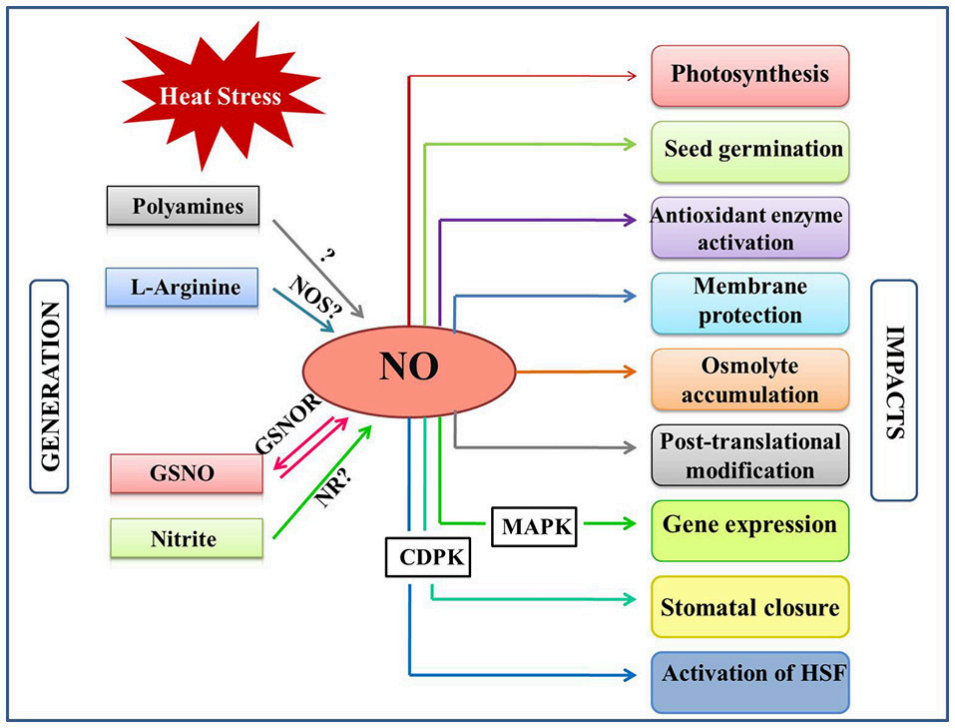
Overview of the NO generation and impacts in plants under heat stress. Heat stress induce NO accumulation majorly by a non-identified NOS-pathway, nitrite-dependent pathway. NO can also be generated by polyamines and by reversible regulation of S-nitrosoglutathione (GSNO) by the action of GSNO reductase (GSNOR). Besides, NO levels are also influenced by various plant hormones under heat stress. This figure also indicates the major plant functions that are mediated by NO-generation/signaling when exposed to heat stress. CDPK, Calcium dependent protein kinase; GSNO, s-ntrosoglutathione; GSNOR, GSNO reductase; HSFs, Heat shock factors; MAPK, mitogen activated protein kinase; NO, nitric oxide; NOS, Nitric oxide synthase; NR, nitratereductase.

Nitric oxide (NO), a free radical gaseous molecule has been shown to be involved in diverse biological functions in plants (Domingos et al., 2015). Being a small diatomic molecule with short half-life, and absence of charge qualifies NO as an ideal diffusible molecular messenger in plant signaling (Yu et al., 2014). Although, initial discoveries in plants recognized NO as an atmospheric toxic pollutant for plant foliage (Wellburn, 1990), it was eventually considered as a modulator of plant defense during pathogen attacks (Durner et al., 1998). In due course, accumulated evidence suggested its key role in diverse plant physiological processes including seed germination (Bethke et al., 2007; Popova and Tuan, 2010), plant maturation and senescence (Mishina et al., 2007), multiple abiotic (Gould et al., 2003; Neill et al., 2008; Molassiotis et al., 2010; Tossi et al., 2012; Sehrawat et al., 2013; Ziogas et al., 2013), and biotic stress responses in plants (Durner et al., 1998; Schlicht and Kombrink, 2013). During the past decade number of studies focused on describing the crucial role of NO in moderating various plant hormone-mediated development and stress responses (Pagnussat et al., 2002; Gould et al., 2003; Freschi, 2013; Asgher et al., 2016). Further, accumulation of NO has been shown to induce gene expression of defense proteins during stress episodes and recovery (Romero-Puertas et al., 2013; Fancy et al., 2017). Mounting evidence suggests the role of NO in maintaining cellular homeostasis by acting as an antioxidant and negating the intensity of oxidative damage caused by various stress treatments (Sang et al., 2008; Karpets et al., 2011; Hasanuzzaman et al., 2012; Groß et al., 2013). Since the functional roles of NO in plants are being explored in parallel under various environmental challenges, it is imperative to recapitulate the current status of research with respect to individual stress conditions to have more focused future investigations addressing gaps in NO-mediated stress signaling in plants. During the past decade, the implication of NO in the mechanism of heat tolerance has been reported in many plant species (Figure 1; Uchida et al., 2002; Song et al., 2006). Despite the growing knowledge about NO-mediated plant heat responses such as decreasing ROS levels by the stimulation of antioxidants, protecting membranes from damage, osmolyte accumulation, and regulation of various hormone–mediated signaling events, its functional status has been far from clarity. Therefore, this review will focus on the current state-of-the-art regarding the emerging functions of NO during plant acclimation to heat stress.

## NO in connection with heat stress

The literature describes an increase in NO accumulation in various plant species in response to high temperature treatments (Leshem, 2001; Yu et al., 2014). Although, these studies underline the emission of heat induced NO as a vital response for increasing plant adaptation to stress (Bouchard and Yamasaki, 2008; Yu et al., 2014), NO burst depends on the severity and duration of heat treatment. For instance, *Nicotiana tabacum* suspension cells exhibited less NO burst upon heat shock at 35°C compared to cells at 55°C (Locato et al., 2008). Moreover, even a short–term heat treatment at 45°C increased NO levels in leaves of *Medicago sativa* and *N. tabacum* leaf peels (Leshem et al., 1998; Leshem, 2001; Gould et al., 2003). Meanwhile, the possibility of NO release as a generalized stress response has been ruled out and its functional specificity has been corroborated by scavenging endogenous NO levels by 2-4-carboxphenyl-4,4,5,5-teramethyllimidazoline-1-oxyl-3-oxide (cPTIO) that eliminated its beneficial effects under heat stress (Zhao et al., 2004; Song et al., 2006). Exogenous application of NO donors has also been able to reduce heat-induced cellular damage further underlining the involvement of NO in plant heat response (Song et al., 2006; Hasanuzzaman et al., 2013). Diverse cellular sites of NO synthesis have been documented previously under heat stress, mostly based on the fluorescent dyes such as 5,6- diaminofluorescein diacetate (Beard et al., 2012). For instance, heat stress induced NO-fluorescence was observed in diverse cell types in *N. tabacum*, including palisade mesophyll cells, all epidermal cell types, such as guard cells, subsidiary cells, long and short trichomes. However, the first appearance of heat induced NO fluorescence occurred in the plastids, followed by nucleus and subsequently spread across the cytosol (Gould et al., 2003). Other than 5,6- diaminofluorescein, electron paramagnetic resonance and chloroflurosence measurements have also been deployed to determine NO levels in thylakoid membrane complexes of *Pisum sativum* (Corpas et al., 2008; Wodala et al., 2008). Nonetheless, few reports contravene this heat induced NO accumulation in plants. Although, in *M. sativa*, heat treatment at 37°C for 2 h resulted in great NO emission, a reduced NO content was observed in *P. sativam* leaves and *Chrysanthemum morifolium* seedlings exposed at 38°C for 4 h (Corpas et al., 2008; Chaki et al., 2011). Furthermore, the reduced NO levels in *C. morifolium* seedlings was followed by a sequence of events such as reduced S- nitrasoglutathione reductase (GSNOR) activity, accumulation of SNOs, an increase in peroxynitrites and consequently intensified protein tyrosine nitration (Chaki et al., 2011). Similarly, heat stress at 38°C for 16 h reduced NO accumulation in the guard cell protoplasts of tree *Nicotiana glauca* (Beard et al., 2012). Abolishing internal NO levels by the treatment with a well- known NO synthase (NOS) inhibitor, L-N(G)-nitroarginine methyl ester, also resulted in similar effects of heat stress in these cells. A comprehensive list of heat stress studies related to NO are provided in Table 1. Although, it is clear that the modulation of intracellular NO levels are crucial under heat stress, the contradictory effects might depend on the type of plant tissues or species and severity of heat treatment.

**Table 1:** Various studies describing the involvement of nitric oxide (NO) in plant heat stress.

**Plant species**	**Heat stress**	NO^*^ donor	**Scavenger/inhibitor**	**Observations**	**Method of NO detection**	**References**
*Lactuca sativa*	27–35°C	0.1–05 mM SNP^*^ 0.1 mM SNAP^*^ 1 mM Fe(III)CN	0.1 mM cPTIO^*^, 0.001–0.2 mM PTIO^*^	NO promotes seed germination, de-etiolation but inhibits hypocotyl, and internodal elongation	ND^*^	Beligni and Lamattina, 2000; Deng and Song, 2012
*Triticum aestivum*	25–43°C	0.05-0.5 mM SNP	0.005 mM Methylene blue	Exogenous NO donor protects from high temperature induced oxidative stress by upregulation of antioxidant defense, methylglyoxal detoxification system and by reducing lipid peroxidation	ND	Karpets et al., 2011; Bavita et al., 2012; Hasanuzzaman et al., 2012; El-Beltagi et al., 2016
*Phragmites communis*	45°C	0.05–0.5 mM SNP & SNAP	0.4 mM cPTIO	Exogenous NO donor confers heat tolerance by promoting active oxygen scavenging enzymes	Spectrophotometry	Song et al., 2006
*Arabidopsis thaliana*	45°C	NA	0.5 mM cPTIO	High endogenous NO inhibits seed germination; stimulates DNA-binding activity of heat shock transcription factors and the accumulation of HSP^*^s by acting upstream of AtCaM3^*^	Fluorescent microscopy DAF-2DA, Chemiluminisence	Hossain et al., 2010; Xuan et al., 2010
*Fragaria ananassa*	42°C	NA	NA^*^	Heat shock treatment increases NO levels, exogenous H_2_S donor induces HSP accumulation in NO dependent manner	Colorimetric assay	Christou et al., 2014
*Oryza sativa*	50°C	0.001–0.02 mM SNP	0.1 mM cPTIO	Exogenous SNP at low levels decreased the deleterious effects of heat stress, alleviated ion leakage, NO acts as an signal molecule in stress tolerance	Spectrophotometry	Uchida et al., 2002; Song et al., 2013
*Phaseolus radiatus*	45°C	0.15 mM SNP	4M Bovine hemoglobin	NO donor promote leaf photochemical activity and cell membrane integrity, increase anti-oxidant enzyme activities thereby eliminating oxidative damage under heat stress	ND	Yang et al., 2006
*Nicotiana tabacum*	35–55°C	1 mM DETA^*^ &, SNP, 0.2 mM NOC-9^*^	0.5, 1 mM cPTIO	Heat stress at 55°C resulted in rapid surge in NO while no detectable increase in NO was observed at 35°C; GSNOR^*^ regulates cellular nitrosation levels by metabolizing GSNO^*^ in plant cells	Fluorescence and confocal microscopy	Gould et al., 2003; Lee et al., 2008; Locato et al., 2008
*Zea mays*	48°	0.05–0.4 mM SNP	NA	Pretreatment with SNP improved the survival percentage of maize seedlings, alleviated electrolyte leakage and lipid peroxidation	ND	Li Z. G. et al., 2013
*Festuca arundinacea*	44°C	0.1 mM SNP	0.2 mM PTIO + 0.2 mM L-NAME^*^	Exogenous NO donor upregulate the transcription of the PSII^*^ core proteins, Psb complex subunits and photosynthesis recovery under heat stress	ND	Chen et al., 2013
*Gingiber officinale*	38°C	0.1 mM SNP	NA	Heat induced effects on relative water content, chlorophyll content, electrolyte leakage were reduced by SNP treatment	Fluorescent microscopy	Li X. et al., 2013
*Helianthus annuus*	30–38°C	NA	NA	Heat stress augments SNO^*^s, tyrosine nitration and inhibits ferridoxin NADP-reductase	Chemiluminisence	Chaki et al., 2011
*Chrysanthemum morifolium*	45°C	0.2 mM SNP	NA	SNP treatment alleviated heat stress by slowing down the reduction of photosynthetic pigment content and net photosynthetic rate	Colorimetric assay	Yang W. et al., 2011
*Pisum sativum*	30°C	NA	0.2 mM cPTIO, 5 mM L-NAME	Heat stress increases NOS^*^ mediated NO production, S-nitrosothiol content and GSNOR activity.	Confocal microscopy, chemoluminiscense, spectrophotometry	Corpas et al., 2008
*Nicotiana glauca*	38°C	2.5 mM SNAP	0.01 mM PTIO, 1 mM L-NMMA^*^	NO is required for auxin-mediated gene expression and cell cycle re-entry in cells under heat stress; Reduced auxin signaling led to reduced ethylene production thereby more cells remained in quiescent state	ND	Beard et al., 2012
*Solanum lycopersicum*	44°C	0.1 mM SNP	NA	SNP along with Ca^+2^ increased Chlorophyll content, osmolytes, total soluble carbohydrate, calcium content; enzyme activities of RuBisCo, carbonic anhydrase, nitrate reductase and anti-oxidant enzymes; decreased MDA^*^ content and chlorophyll degradation	ND	Siddiqui et al., 2017
*Citrus aurantium*	42°C	NA	0.3 mM cPTIO	A significant increase in the NO and S-nitrosylated protein content; reduced expression of AOX^*^ and NiR^*^ genes	Confocal microscopy	Ziogas et al., 2013

* *AOX, Alternative oxidase; AtCaM3, Arabidopsis thaliana Calmodulin 3; cPTIO, 2-4-carboxphenyl-4,4,5,5-teramethyllimidazoline-1-oxyl-3-oxide; DAF-2DA, 4,5-Diaminofluorescein diacetate; GSNO, S- nitrasoglutathione; GSNOR, S- nitrasoglutathione reductase; HSP, Heat shock proteins; H_2_S, Hydrogen sulfide; PTIO, 2-phenyl-4, 4, 5, 5,-tetramethylimidazoline-1-oxyl 3-oxide; L-NAME, N Nitro-L- aginine methyl ester; MDA, melanoldialdehyde; NiR, Nitrite reductase; NA, Not Applicable; ND, Not detected; NO, Nitric oxide; NOS, nitric oxide synthase; SNAP, S-nitrose-N-acetyl penicillamine; SNO, s-nitrosothiols SNP, sodium nitroprusside*.

## NO synthesis and signaling in plant heat stress

Despite evidence on functional existence of NO in plants, its metabolic origin and mode of its involvement in signaling pathways is yet unresolved (Gupta et al., 2011). Some of the pathways in NO production include NOS pathway (Delledonne, 2005; Negi et al., 2010), nitrate reductase (NR) pathway (Morot-Gaudry-Talarmain et al., 2002), other enzymatic and non-enzymatic pathways (Gupta et al., 2011). Although, the source of heat stress-induced NO accumulation is not clearly understood, few studies have reported an L-arginine-dependent production of NO through NOS-like activity (Corpas et al., 2008; Wodala et al., 2008) and NR-mediated production (Siddiqui et al., 2017). However, other studies demonstrated heat-induced accumulation of NO rather independent of enzymes (Hancock, 2012). NOS-dependent increase in the NO production was observed in Zooxanthellae (*Symbiodinum microadriaticum*), a marine microalgae, in a temperature-dependent manner (Bouchard and Yamasaki, 2008). Similarly, heat stress-induced increase in endogenous NO production in mycelial cells of edible fungi (*Pleurotus eryngii*) was found sensitive to the NOS inhibitor nitro-L- aginine methyl ester (L-NAME), while the NR suppressor tungstate had no effect, suggesting a NOS-dependent response to heat stress (Kong et al., 2012). High temperature induced NOS and GSNOR activities followed by the accumulation of NO and S-nitrosothiols without much change in the concentration of nitrites and nitrates in *P. sativum* leaves (Wodala et al., 2008). While this suggests that NOS activity is responsible for the NO produced in *P. sativum* leaves, the possibility of NO production from sources other than NOS activity cannot be ruled out.

While the source of NO under high temperature is yet to be defined in plants, the endogenous levels of NO in plant cells is regulated by many other factors such as S- nitrasoglutathione (GSNO) (Leterrier et al., 2012). This GSNO is a natural reservoir of NO, being transported over long distances through the phloem. The turnover of GSNO has been known to be controlled under stress conditions by GSNOR1, impacting the NO hemostasis in plant cells (Liu et al., 2001; Cheng et al., 2015). The *A. thaliana* loss-of function mutations in *sensitive to hot temperatures 5 HOT5 GSNOR1* locus resulted in high heat sensitivity and increased s-nitrosothiols (SNO) and nitrate concentrations (Larkindale et al., 2005; Crawford et al., 2006; Lee et al., 2008). Moreover, heat sensitivity in these mutants was rescued using a NO scavenger (Lee et al., 2008). While the mutation in *NOA1* locus encoding a GTPase, *CUE1* gene encoding a chloroplast phosphoenolpyruvate/phosphate translocator, *ARGAH1* or *ARGAH2* encoding arginine amidohydrolase-1 and -2, respectively, *PHB3* gene encoding prohibition are known to regulate NO levels in *A. thaliana*, their heat stress responses need further exploration (Flores et al., 2008; Lee et al., 2008; Moreau et al., 2008; Wang et al., 2010; Fröhlich and Durner, 2011). Besides, generation of peroxynitrite (ONOO^−^) from the reaction between NO^·^ and ${\text{O}}_{2}^{\cdot }$ and NO dioxygenase activity that produces ${\text{NO}}_{3}^{-}$ from NO and ${\text{O}}_{2}^{\cdot -}$ also contribute for the elimination of NO from plant cells (Sanz-Luque et al., 2015). Good progress has been made in the recent years toward understanding the post-translational modifications (PTMs) mediated by NO in plants such as protein S-nitrosylation and tyrosine nitration, that have immense influence in the activity of various enzymes that confer stress tolerance (Chaki et al., 2011; Romero-Puertas et al., 2013; Fancy et al., 2017).

## Heat amelioration by exogenous NO donors

Exogenous NO donors have been often deployed successfully as priming agents to ward off abiotic stress induced losses in plants (Uchida et al., 2002; Zhao et al., 2007; Sang et al., 2008; Hasanuzzaman et al., 2012; Santisree et al., 2015; Savvides et al., 2016). Different donors/inhibitors of NO have been deployed in plant heat studies using various application methods (Tables 1, 2). In *Triticum aestivum* seedlings, pretreatment with 0.25 mM sodium nitroprusside (SNP) enhanced ascorbate and glutathione contents and activities of monodehydroascorbate reductase, dehydroascorbate reductase, and glyoxalase I and II under heat stress at 38°C given for 48 h (Hasanuzzaman et al., 2012). Likewise, *Zea mays* seedlings pretreated with 0.15 mM SNP enhanced the heat stress survival percentage at 48°C for 18 h as a result of reduced electrolyte leakage and malondialdehyde (MDA) content (Li Z. G. et al., 2013). The extent of its impact depends on factors like timing of application, developmental stage of the plant, combination of stress strength and donor concentration, tissue analyzed, and plant species (Chaki et al., 2011). Usually, exogenous application of selected dose of NO donors is a cost-effective approach in protecting plants from heat stress. However, it is very essential to establish the suitable NO donor, dosage, toxicity, complete NO releasing mechanism, byproducts and their bioactivities before large scale agricultural applications (Santisree et al., 2015). While, the use of SNOs, such as GSNO, the natural reservoir of NO in plants, could be encouraged under heat stress conditions, the release of NO from donors such as SNP often relies on the environmental factors like light, temperature, pH of the cells etc. and released toxic byproducts need to be carefully considered. Although, it is difficult to apply and measure the real cellular concentrations of NO donors/inhibitors, this pharmacological approach seems inevitable until the complete elucidation of NO generation in plants. In spite of technical difficulties, exploration of specific targets of these donors (Santisree et al., 2017a), cautious interpretation of results and combination of genetic and pharmacological approach seems more promising to give insights into NO-mediated heat stress regulation in future.

**Table 2:** Various methods deployed for exogenous nitric oxide treatment in plant heat stress studies.

NO^*^ loading	**NO Donor**	**Plant species**	**Response**	**References**
Imbibition	SNP	*Lactuca sativa, Triticum aestivum, Arabidopsis thaliana*	Imbibition of seeds with SNP resulted in enhanced seed germination and increased activity of β-amylase; Application of SNP, Fe(III)CN and acidified nitrite reduced thermo-inhibition in *Lactuca sativa* seed germination in an NO-dependent mechanism, while cPTIO reversed this process	Beligni and Lamattina, 2000; Bavita et al., 2012; Deng and Song, 2012
Hydroponics	SNP	*Triticum aestivum, Oryza sativa*	Hydroponic treatment with SNP prior to germination showed alleviated heat stress symptoms such as increase in active oxygen scavenging enzymes activities, anti-oxidant enzymes	Uchida et al., 2002; Hasanuzzaman et al., 2012
Foliar spray	SNP	*Arabidopsis thaliana, Triticum aestivum, Chrysanthemum morifolium*	Foliar spray of SNP lowered the heat-induced increase in the non-photochemical quenching, malondialdehyde content, maintained higher activities of superoxide dismutase, peroxidase, catalase and ascorbate peroxidase; NO improved survival under high heat and acts upstream of AtCaM3 in acquiring thermotolerance	Xuan et al., 2010; Karpets et al., 2011; Yang W. et al., 2011
Supplemented in the growth medium	SNP & SNAP	*Triticum aestivum, Phargmites communis*	Supplementing NO in the growth medium protects against oxidative stress induced by heat stress by increasing the content of non-enzymatic and enzymatic anti-oxidants and relative cell viability; alleviates ion leakage, H_2_O_2_ content; decrease of tiol content	Song et al., 2006; El-Beltagi et al., 2016
Pre-soaking	SNP	*Oryza sativa, Phaseolus radiatus*	Pre-soaking leaf discs in SNP increases thermotolerance by activating oxygen scavenging enzymes and reducing lipid peroxidation	Yang et al., 2006; Song et al., 2013
Vacuum Infiltration	SNP	*Festuca arundinacea*	Vacuum infiltration improves the photosynthesis, heat stress recovery process of PSII by increasing the expression of PSII core proteins	Chen et al., 2013
Irrigation	SNP	*Gingiber officinale*	SNP irrigation increased the leaf relative water content, chlorophyll content and reduced electrolyte leakage	Li X. et al., 2013

## NO as antioxidant

Heat stress induces an increased generation of reactive oxygen species (ROS) such as the superoxide anion radicle (${\text{O}}_{2}^{\cdot -}$) and hydrogen peroxide (H_2_O_2_) resulting in oxidative stress that causes a disturbance in the redox balance of cells (Khan et al., 2013a). These free radicals damage membranes and macromolecules that subsequently have detrimental effects on plant metabolism and yield (Santisree et al., 2015; Awasthi et al., 2016). Thus, the faster removal of ROS by antioxidant enzymes or molecules is often the deciding factor for plant survival under heat stress condition. The antioxidant function of NO has been well-documented under a wide range of stress responses including heat stress (Beligni and Lamattina, 1999; Uchida et al., 2002; Tian et al., 2007; Song et al., 2008; Palmieri et al., 2010; Santisree et al., 2015). Two significant roles of NO has been established in mitigating heat induced oxidative stress response, one is the maintenance of cellular redox homoeostasis due to its ability to neutralize the harmful ROS, and another is NO-induced protection against oxidative stress through moderating the carotenoids under heat stress. Carotenoids are known as protecting agents against photo-oxidative damage due to their ability to neutralize free radicals. Hence, enhanced levels of carotenoids offer better protection under heat stress. Foliar application of SNP enhanced the carotenoid content in heat-stressed *C. morifolium* which can be attributed to enhanced heat tolerance (Yang W. et al., 2011).

The most demonstrated ability of NO in reducing ROS levels is by enhancing antioxidant enzyme activities under unfavorable temperatures (Neill et al., 2002; Karpets et al., 2011). For instance, stimulation of ascorbate peroxidase and glutathione reductase enzyme activities by heat stress-induced NO signal in *T. aestivum* plays an important role in the metabolism of H_2_O_2_ (El-Beltagi et al., 2016). Likewise, 0.5 mM SNP-activated antioxidant enzymes superoxide dismutase, catalase and soluble peroxidase, were positively correlated with elevated tolerance in *T. aestivum* coleoptiles heat shocked at 43°C for 10 min (Karpets et al., 2011). Exogenous application of NO donor also conferred heat tolerance to the seedlings of *Phaseolus radiatus* and *Phragmites communis* by activating scavenging enzymes (Yang et al., 2006; Song et al., 2008). However, pretreatment of young *O. sativa* plants with 1 μM SNP alleviated salt or heat stress while high concentrations of SNP (>100 μM) decreased growth rate (Uchida et al., 2002). Promotion of antioxidant activity during heat stress period is the common NO-mediated protective response observed in most species, nonetheless there are some cases where NO sustained the antioxidant defense even after the stress episode. For instance, treatment with SNP not just enhanced heat-induced antioxidant enzymes activities, but also maintained their level during later stages as well in heat-stressed *C. morifolium* (Yang W. et al., 2011). Treatment of *T. aestivum* coleoptiles with scavenger of superoxide reduced ${\text{O}}_{2}^{\cdot -}$ generation and reversed heat resistance induced by SNP (Karpets et al., 2011). This suggests the importance of superoxide generation in SNP-induced heat resistance. However, prolonged and much sever heat stress episodes lead to the nitrosative stress due to substantially increased SNO content in *P. sativum* (Corpas et al., 2008). Hence, maintaining cellular NO status is essential to acquire thermotolerance in plants. GSNOR regulates endogenous NO levels by metabolizing GSNO, which is a well-known cytoplasmic NO reservoir in both plants and animals (Liu et al., 2001). The absence of GSNOR undeniably affects NO/nitroso levels and subsequently affects heat sensitivity. Hence, it was demonstrated that GSNOR function is required for acclimation to high temperature and for normal plant growth and fertility (Lee et al., 2008; Xu et al., 2013). The importance of GSNOR has been highlighted by a null mutation (*atgsnor1-3/hot5-2*) or RNAi line that reduced *GSNOR* expression and lead to over-accumulation of NO that subsequently correlated with the heat-sensitive phenotype in *A. thaliana*. Moreover, these thermotolerance defects has been rescued with NO scavengers further supporting the link between excess NO-related nitrosation and plant heat sensitivity in plants (Lee et al., 2008). Apart from this, NO overproducing *nox1* mutant seedlings were defective in heat tolerance while, *noa1* and *nia1/nia*, mutants producing less endogenous NO (Crawford et al., 2006) were similar to wild-type seedlings in their heat tolerance. Although, the available information made it clear that maintaining cellular levels of NO is an important component of plant heat stress response, the fine regulation of ROS/NO using exogenous NO donors under heat stress needs further investigations.

## NO and membrane integrity

One of the important components of heat tolerance is the maintenance of membrane integrity. Heat stress induced oxidative burst significantly increase the membrane lipid peroxidation consequently reduce the membrane thermostability and increase electrolyte leakage in plants (Hasanuzzaman et al., 2013; Khan et al., 2013a). By virtue of its free radical nature, NO may interact with lipid hydroperoxyl or superoxide radicals that promote lipid peroxidation and acts as a chain breaker to ensure the protection of membranes (O'Donnell et al., 1997). It was found that application of 0.2 mM SNP and S-nitrose-N-acetyl penicillamine dramatically reduced heat induced ion leakage, reduction in growth, and loss of cell viability in callus of *P. communis* treated with 45°C for 2 h (Song et al., 2006). High temperature causes enhanced accumulation of thiobarbituric acid reactive substances by reducing membrane protein thiol level (Bhattacharjee, 2009). While the thiobarbituric acid reactive substances content was substantially reduced in 0.15 mM SNP-treated *P. radiatus* leaf discs, that was partially reversed by adding equal concentration of NO scavenger, thereby suggesting NO-induced protection from lipid peroxidation (Yang et al., 2006). 0.2 mM SNP also lessened lipid peroxidation in the leaves of *C. morifolium* plants subjected to 45°C for 24 h (Yang W. et al., 2011). Reduced lipid peroxidation by SNP treatment was also evident by the reduction in membrane leakage by up to 48% in comparison with the non-treated controls in *P. radiatus* leaf discs (Yang et al., 2006). Similarly, alleviation of heat-induced lipid peroxidation and electrolyte leakage by pretreatment with SNP was also demonstrated in *T. aestivum* seedlings (Hasanuzzaman et al., 2012). Heat stress-induced MDA content in hydroponically grown *T. aestivum* seedlings was brought down substantially by SNP treatment (Hasanuzzaman et al., 2012). Moreover, enhanced membrane thermostability and reduced lipid peroxidation by NO were more pronounced in the shoots over roots of heat tolerant *T. aestivum* that could suggest site-specific regulation of NO effects under heat stress (Bavita et al., 2012). Similarly in another study, exogenous application of SNP recovered relative water content, chlorophyll(chl) concentration, electrolyte leakage in heat stressed *Gingiber officinale* leaves consequently normalized chl fluorescence parameters (Li X. et al., 2013). Taken together, these studies provided good evidence that NO donor treatment can enhance membrane stability and thus reduce electrolyte leakage under heat stress.

## NO and photosynthesis under heat stress

Photosynthesis, a vital plant process that forms sole basis for all assimilates is highly vulnerable to heat stress (Allakhverdiev et al., 2008; Khan et al., 2013b; Mathur et al., 2014). High temperature affects the physicochemical properties and functional organization of thylakoid membrane thus irreversibly damaging the chloroplast protein complexes including photosystem II (PSII) (Brestic et al., 2012). Heat stress also induce some reversible effects such as increase in photorespiration, decreased activities of Calvin–Benson cycle enzymes including RuBisCo, RuBisCO activase. Both reversible and irreversible effects of heat often lead to yield penalty as well as longer impacts on plant metabolism (Brestic et al., 2014, 2016; Mathur et al., 2014). Hence, increasing photosynthetic efficiency by enhancing heat acclimation of photosynthetic apparatus is one of the most desirable future target (Yamori et al., 2014). To achieve this goal, it is vital to understand the processes that limit the photosynthetic productivity under heat stress and the role of NO in ameliorating these processes.

Plants show reduced chl biosynthesis and damage of photosynthetic apparatus such as swelling of grana stacks followed by ion leakage form leaf cells when exposed to high temperatures (Allakhverdiev et al., 2008). Several studies suggest the role of NO in heat acclimation of photosynthesis at various levels. In several species such as *C. morifolium, Quercus pubescens, O. sativa, Vicia faba*, and *Solanum lycopersicon*, heat stress induced decrease in photosynthesis rate (Haldimann and Feller, 2004) and chl bleaching (Misra, 1980, 1981; Misra et al., 1997; Brestic et al., 2012) got partially alleviated by SNP treatment by delaying the reduction of photosynthetic pigment content and net photosynthetic rate (Yang Q. et al., 2011). Literature suggests the inactivation of both electron acceptor and donor sides of PSII by high temperature (Pospíšil and Tyystjarvi, 1999; Mathur et al., 2011, 2014; Brestic et al., 2012). NO has been shown to prevent this heat induced chl loss and maintain the activity of PSII, thereby mitigating the reduction in photosynthesis (Misra, 1981). SNP treatment also helped to retain the level of Fv/Fm and inhibited the rise in F_*o*_ (Yang Q. et al., 2011). As an evidence, pre-treatment with 100 μM SNP resulted in enhanced photosynthetic electron transport in *Festuca arundinacea* at 44°C (Chen et al., 2013). Similarly, increased survival rate of *T. aestivum* and *Z. mays* seedlings was also observed under extreme temperatures due to SNP treatment. The involvement of NO in heat acclimation of photosynthesis was further confirmed by the temperature-dependent synthesis of chl as well as RuBisCo by overexpressing *NOA1* in *O. sativa* plants (Yang Q. et al., 2011). In addition to its role in enhancing chl biosynthesis, NO is also shown to reduce chl degradation (Kong et al., 2014). The reduced chl loss in NO treated plants can also attribute to its close relation to iron metabolism which has a direct correlation with chl biosynthesis and plant productivity. It has been observed earlier that exogenous application of SNP promoted uptake, translocation and internal availability of iron in plants (Graziano et al., 2002; Zhang et al., 2012; Kong et al., 2014). In addition, exogenous NO donor was also known to revert the chlorotic phenotype of *Z. mays* mutants *yellow stripe1* and *yellow stripe3* defective in iron uptake mechanism providing genetic evidence for its role in NO uptake(Graziano et al., 2002). Furthermore, heat-induced structural and functional changes in the thylakoid membrane often results in ROS formation (Pospíšil et al., 2007; Pospíšil and Prasad, 2014; Pospíšil, 2016). Heat stress induced oxidative stress slowdown carbon assimilation by inhibiting the activities of ferredoxin–NADP oxidoreductase and carbonic anhydrase by 31 and 43%, respectively. Indeed, the antioxidant property of NO has been demonstrated by many studies where NO acts as a signal to induce the antioxidative enzyme activity, consequently protect plants from heat-induced oxidative damage (Song et al., 2008). Besides, NO has been shown to influence reversible inhibition of key enzymes of photosynthesis by tyrosine nitration (Chaki et al., 2011, 2013). For instance, the combined application of SNP and calcium resulted in higher levels of *Chl a* and *b* due to reduced Chl degradation, and also enhanced activities of RuBisCo, carbonic anhydrase and NR under heat stress in *Solanum lycopersicum* (Siddiqui et al., 2017). Non-photochemical quenching is a mechanism of protection to the photosynthetic apparatus by dissipating excess energy, while also reducing the excitation energy necessary for PSII execution (Gilmore, 1997). Treatment with 5 mM SNP reduced the rate of Non-photochemical quenching in *T. aestivum* leaf discs heat shocked for 2 h at 35°C and thus diverted more energy to PSII (Hossain et al., 2011). Similarly, a T-DNA insertional mutant of *Arabidopsis thaliana glb3* that has knocked-out for class 3 hemoglobin gene responsible to scavenge excess NO, exhibited severe heat induced reduction in non-photochemical quenching due to over accumulation of NO (Hossain et al., 2011). While this was completely prevented by cPTIO, exogenous NO donors photocopied the observed response. These results clearly demonstrate the role of NO in heat induced decline of non-photochemical quenching (Hossain et al., 2011).

Despite of its protective effects on photosynthesis uncontrolled burst of NO due to high temperatures results in damage to the photosynthetic machinery (Zhang et al., 2009). Excess NO inhibits electron transport by reversibly binding to thylakoid membrane complexes of *P. sativum* (Wodala et al., 2008). Similarly, the levels of NO, S-nitrosothiols (SNO), and ${\text{O}}_{2}^{\cdot -}$ were also enhanced leading to nitrosative stress under high heat in *Citrus aurantium* plants (Ziogas et al., 2013). This increment is associated with reduced chl content and increased electrolytic leakage. In *Symbiodinium microadriaticum*, heat-induced NO production caused a decline in the photosynthetic efficiency of PSII (F_x_/F_m_), which correlated with the coral bleaching phenomenon (Bouchard and Yamasaki, 2008). However, unlike other free radicals, the generated NO can diffuse rapidly. In a situation like heat stress where photosynthetic electron transport is restricted, the cytosolic nitrite is converted to NO by NR in presence of NAD(P)H and diffuses out easily into the atmosphere (Yamasaki, 2000). Hence, the emission of NO has been speculated as an effective way to dissipate excess free radicals under heat stress.

## Seed germination and osmolyte accumulation

Although, slightly higher than optimum growth temperature is needed for seed germination, heat stress exerts negative impact on seed germination (Essemine et al., 2007; Essamine et al., 2010; Brunel-Muguet et al., 2015). High temperature affects the rate of germination, percentage of germination, seedling emergence in many plant species. At times heat stress completely prohibited seed germination due to embryo damage and cell death (Johkan et al., 2011). Data have shown the promotion of seed germination by exogenous application of NO-releasing compounds in various plants (Bethke et al., 2006; Arc et al., 2013). NO has been implicated in promotion of seed germination in many species such as *A. thaliana, Hordeium vulgare, Panicum virgatum, Lactuca sativa, Stellaria media, Malus domestica* either by reducing seed dormancy, or by reducing the effects of adverse environmental conditions (Giba et al., 1992; Beligni and Lamattina, 2000; Zhang et al., 2005; Bethke et al., 2007; Krasuska et al., 2017). One mechanism of reduced seed germination by heat stress is by decreasing the mobilization and utilization efficiency of seed reserves (Blum and Sinmena, 1994). SNP has been found to promote seed germination by inducing the activity of β-amylase during early stages of seed germination in many plant species such as *A. thaliana, T. aestivum, M. sativa* (Zhang et al., 2005; Duan et al., 2007; Maurice et al., 2016). NO has been implicated in loss of dormancy by decreasing seed sensitivity to abscisic acid (ABA), while acting upstream of gibberellic acid during vacuolation of protein storage vacuoles (Bethke et al., 2006, 2007). However, according to a recent study excessive NO production under heat stress is speculated to be involved in thermo-inhibition of seed germination in *A. thaliana* (Hossain et al., 2010). Further, the seeds of *A. thaliana* T-DNA insertion mutant *glb3* compromised in NO scavenging activity, fail to remove heat-induced excessive NO and hence, show high temperature sensitivity during germination at 32°C, while NO scavenger cPTIO partially restored the germination up to 40% in comparison with 100% in the wild type (Hossain et al., 2010). Similarly, application of SNP, Fe(III)CN, and acidified nitrite reduced thermo-inhibition in *L. sativa* seed germination in an NO-dependent mechanism, while cPTIO reversed the stimulation of seed germination by these compounds (Deng and Song, 2012). Although, the optimum temperatures for *L. sativa* seed germination were 11–19°C in light, SNP promoted germination up to 25°C in light in a dose-dependent manner (Deng and Song, 2012). Not only that, seed germination of *L. sativa* seeds at 25°C was independent of light in NO donor imbibed seeds (Beligni and Lamattina, 2000). Based on these results the relevance of NO emission within the deeper soil layers has been speculated to stimulate light responses such as germination where photoperception is low being even more potent than gibberellin. This notion was supported by the involvement of NO in the phytochrome controlled seed germination in *Paulownia tomentosa* (Giba et al., 1998). Seed dormancy due to soil temperature has been recently linked to the regulation of ABA metabolism and signaling genes (Arc et al., 2013). Indeed, *A. thaliana* copper amineoxidase, *cuo1* mutant seedlings defective in ABA-induced NO synthesis, exhibit a reduced sensitivity to exogenous ABA during germination (Wimalasekera et al., 2011).

Several lines of evidence have reinforced the importance of accumulation of osmolytes such as proline, glycine-betaine, and soluble sugars to maintain osmotic balance in heat stressed plants (Hasanuzzaman et al., 2013). SNP pre-treatment up-regulated the *P5CS* gene in *O. sativa* seedlings that helped in survival after exposition to heat stress (Uchida et al., 2002). Similarly, transcriptional induction of *P5CR* by heat and salt stress was observed in *A. thaliana* (Hua et al., 2001). Indeed, a 92 bp fragment of the *P5CR* 5′UTR was found as a regulator to control its mRNA stability under heat stress (Hua et al., 2001). The combined application of SNP along with calcium enhanced proline as well as glycine-betaine, osmolyte content observed in heat-stressed *S. lycopersicum* seedlings (Siddiqui et al., 2017). From previous studies, it is known that while exogenous application of polyamines to *A. thaliana* seedlings induced NO production, exogenous application of NO donor also promoted polyamine metabolism in plants. For example, SNP treatment reduced putricine/PAs ratio in *G. officinale* leaves under heat stress (Li X. et al., 2013). Global metabolite profile of NO-treated *A. thaliana* seedlings also supported the accumulation of polyamines by NO as a possible strategy to attenuate stress-induced cell death (León et al., 2016). Although, direct link between polyamines and NO is yet to be revealed under heat stress, a general concentration-dependent proline and polyamine metabolite accumulation was reported by SNP treatment in *Medicago truncatula* leaves (Filippou et al., 2013). In fact, increased polyamine biosynthesis by any means can also result in increased proline content (Minocha et al., 2014). Heat stress often decreases soluble sugars and proteins that are also implicated in the loss of fertility due to failed pollen germination in crops such as *C. arietinum* (Kaushal et al., 2013). While exogenous NO enhanced soluble sugars and proteins in various stress responses such as salinity, drought etc., increased soluble sugar content under heat stress is yet to be studied (Ahmad et al., 2016).

## NO regulation at gene and protein level

NO trigger plant responses at multiple levels, from gene to the metabolites to achieve heat stress tolerance (Palmieri et al., 2010; León et al., 2016). Few studies using the mutational and transgenic approaches have provided molecular insights into the role of NO in thermotolerance in plants (Table 3). In mutants *gsnor1/hot5* and *nox1*/*cue1*, unusual thermotolerance was observed due to the accumulation of NO (Fancy et al., 2017). As discussed earlier, *A. thaliana* seeds of *glb3* mutant lacking the hemoglobin gene also accumulated high cellular NO exhibit high temperature sensitivity akin to NO donor treated wild type plants. Although these mutants increased the momentum of NO exploration in heat stress studies, complete characterization of such responses is imperative to elucidate NO role in heat stress. Similarly, transgenic efforts using nNOS lines of *N. tabacum*, antisense suppression of GSNOR or overexpression of non-symbiotic hemoglobins in *A. thaliana* also confirmed the role of NO in heat stress tolerance (Table 3; Lee et al., 2008; Hossain et al., 2010; Shi et al., 2014).

**Table 3:** List of few mutants and transgenic lines used in elucidation of nitric oxide (NO) role in plant heat tolerance.

**Transgenes/Mutants**	**Plant species**	**Gene mutated/overexpressed**	**Response**	**Temperature**	**References**
**MUTANTS**					
*ARGH1&2*	*Arabodopsis thaliana*	Arginine amidohydrolase-1 and -2	Increased accumulation of NO compared to wild type	NA	Flores et al., 2008
*atgsnor1-3/hot5-2*	*Arabidopsis thaliana*	S-nitrosoglutathaione reductase	GSNOR-regulated NO homeostasis, GSNOR function is required for normal plant growth, fertility and acclimation to high temperature	38 & 48°C	Lee et al., 2008
*nia1, nia2*	*Arabidopsis thaliana*	Nitrate reductase	NO functions in signaling, stomatal closure and acts upstream of AtCaM3	45°C	Xuan et al., 2010
*nox-1/cue-1*	*Arabidopsis thaliana*	chloroplast phosphoenolpyruvate/phosphate translocator	Antioxidant and osmolyte levels	25°C	He et al., 2004
*atrbohB, atrbohD*, and *atrbohB/D*	*Arabidopsis thaliana*	NADPH oxidase-defective	NO acts downstream to H_2_O_2_ in signaling	45°C	Wang et al., 2014
**TRANSGENICS**					
ΔAtGLB3	*Arabidopsis thaliana*	T-DNA insertion mutant lacks a functional gene encoding a homolog of bacterial truncated Hb (trHb)	Chemical scavengers for reactive nitrogen species potentially improve seed germination at high temperature	32°C	Hossain et al., 2010
*AtGSNOR1;RNAi*	*Arabidopsis thaliana*	Absence of *Atgsnor1-3* expression	Increased nitrate concentrations	NA	Fröhlich and Durner, 2011
*atrbohB/D/35S*::*NIA2-1* and *atrbohB/D/35S*::*NIA2-3*	*Arabidopsis thaliana*	*AtNIA2*-overexpressing transgenic lines	Increase in the survival ratio of the seedlings, H_2_O_2_ acts upstream of NO in thermotolerance	45°C	Wang et al., 2014
*BA-mgfp5-ER*	*Nicotiana glauca*	Transformed guard cell protoplasts with auxin responsive BA promotor	Plants that have evolved to withstand sustained high temperatures may still be negatively impacted by heat stress	32 & 38°C	Beard et al., 2012
*OsNOA1*	*Oryza sativa*	*Nitric oxide associated1*-suppressed RNAi lines	Temperature-dependent manner to regulate chlorophyll biosynthesis, Rubisco formation and plastid development in rice	22 & 30°C	Yang Q. et al., 2011

During the last decade, few studies demonstrated implications of NO on modulation of gene transcription and post-translational modifications of proteins during stress conditions in plants by using exogenous donors (Ahlfors et al., 2009; Besson-Bard et al., 2009; Palmieri et al., 2010). NO induced transcription of many stress-inducible genes improved the plant survival and yield under heat stress (Begara-Morales et al., 2014). Similarly, SNP pretreatment improved heat stress tolerance in *O. sativa* seedlings by inducing gene transcription of sucrose-phosphate synthase, small heat shock protein (HSP) 26 and d-pyrroline-5- carboxylate synthase (Uchida et al., 2002). Similarly, the expression of *alternative oxidase* and *nitrite reductase* were observed to be reduced in the heat-treated *C. aurantium* plants (Ziogas et al., 2013). While, transcriptomic analysis of GSNO-responsive genes in *A. thaliana* roots showed up-regulation of various heat stress transcription factors and HSPs (Begara-Morales et al., 2014), the heat induced accumulation of Hsp70 corresponded to the endogenous NO level in *S. lycopersicum* leaf discs (Piterková et al., 2013) pointing toward an important role of HSPs in protecting cellular membranes and protein folding and aggregation during heat stress. Another study indicated the NO induced stimulation of *A. thaliana* calmodulin 3(*At*CaM3), which in turn activates its heat shock factor-DNA binding activity and HSP gene expression imparting heat tolerance (Xuan et al., 2010). There have been pointers on NO in a signaling cascade together with other signaling molecules such as ABA, H_2_O_2_, Ca^2+^, and calmodulin, stimulate DNA-binding activity of heat shock factors and abundance of HSPs, leading to heat stress resistance in plants (Khan M. N. et al., 2015).

NO directly alters a wide variety of key proteins through PTMs resulting in remarkable change in their activity, ionic state, aggregation, and cellular location establishing the functional relevance of NO signaling in plants (Martinez-Ruiz et al., 2011). Recent studies demonstrated these PTMs as the most effective means through which NO exerts its global impact on most of the proteins and enzymes during various stress conditions (Romero-Puertas et al., 2013). NO-mediated post-translational modifications such as S-nitrosylation and tyrosine nitration has been involved in heat stress responses in plants (Fancy et al., 2017). Protein tyrosine nitration was intensified by heat stress in *P. sativum* leaves indicating the nitrosative stress caused by high temperatures (Corpas et al., 2008). Similarly, the heat induced nitrosative stress lead to enhanced protein nitration in *C. morifolium* seedlings. Detailed analysis of the nitroproteome revealed the induction of 13 tyrosine-nitrated proteins by heat stress including enzymes like ferredoxin–NADP oxidoreductase and carbonic anhydrase (Chaki et al., 2011). Further, it was found by *in silico* analysis that Tyr^205^ is the most likely potential target for nitration in *P. sativum* CA sequence (Chaki et al., 2013). Similarly there have been reports on NO-induced post-translational modifications in antioxidant enzymes such as catalse, superoxide dismutase, peroxiredoxins, as well as enzymes of the ascorbate-glutathione cycle during stress conditions (Santisree et al., 2015). S-nitrosylation of antioxidant enzymes not just stimulates their activities, but also protects Cys residues from ROS-mediated irreversible oxidations. Similarly, inhibition of ascorbate peroxidase activity by S-nitrosylation also acts as signaling PTM due to subsequent degradation by ubiquitination followed by programmed cell death in *N. tabacum* BY2 cells under heat stress (de Pinto et al., 2013). Several other proteins such as GSNOR and NADPH are also prone to S-nitrosylation, with *S*-nitrosylation of GSH forming GSNO, that in turn mediates transnitrosylation reactions under stress (Lee et al., 2008; Xu et al., 2013). A detailed global molecular profiling by omics approaches could potentially increase this knowledge on the effect of NO and NO-mediated PTMs at subcellular level in order to mitigate nitrosative stress impacts induced by high heat.

## NO and other signaling molecules under heat stress

**Figure 2:**
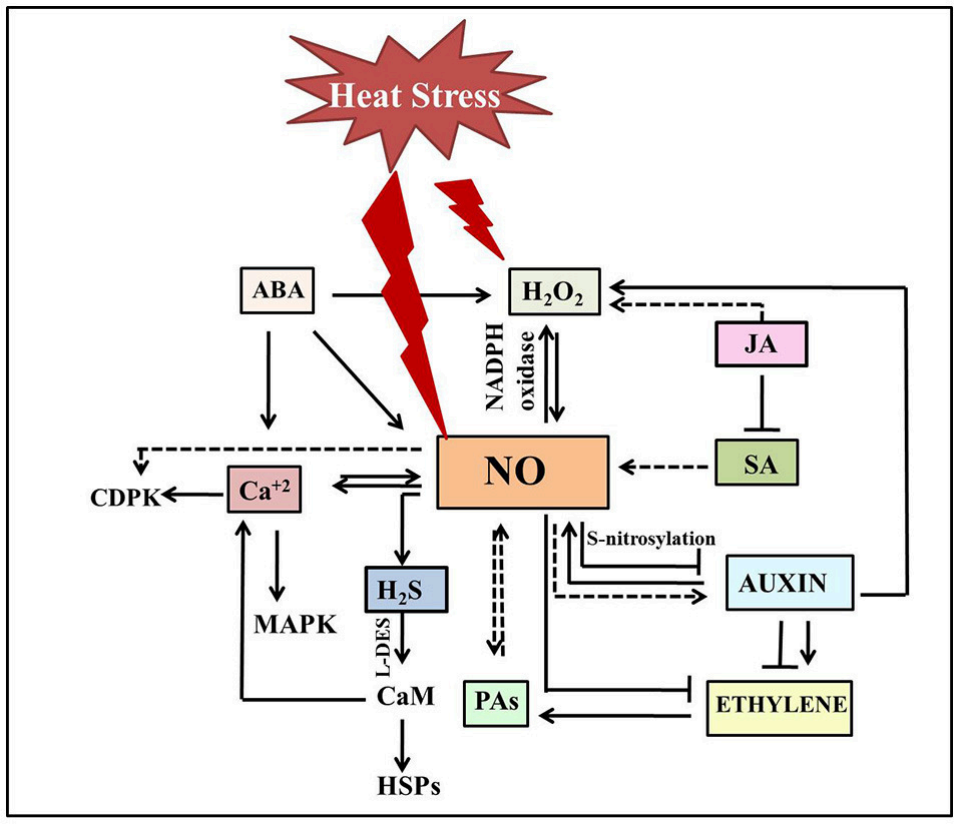
Schematic illustration depicting cross talk of NO with other signaling molecules under heat stress. Heat stress triggers NO accumulation which in turn either stimulates (normal end arrow) or inhibits (blunt end arrow) hormone-mediated heat stress signaling. NO mostly acts downstream to phytohormones under heat stress. Plant heat tolerance not just include crosstalk between H_2_O_2_, NO, and H_2_S, but also involve activation of Ca^2+^ channels, and Ca^2+^-CaM-dependent protein phosphatase along with other factors to induce DNA-binding activity of the heat shock factors and subsequent accumulation of HSPs. The dotted line denotes the pathways not studied clearly. The double-sided arrow indicates the mutual regulation of molecules. ABA, Abscisic acid; Ca^2+^, calcium ion; CaM, calmodulin; CDPK, calcium dependent protein kinase; H_2_O_2_, hydrogen peroxide; HSPs, heat shock proteins; H_2_S, Hydrogen sufide; JA, jasmonic acid; MAPK, mitogen activated protein kinase; NO, nitric oxide; SA, salicylic acid; PAs, polyamines.

NO signaling under heat stress involves a cross talk with other signaling molecules such as cyclic adenosine diphosphate ribose, mitogen activated protein kinases, Ca^2+^, and phytohormones (Figure 2; Mioto et al., 2014; Khan M. N. et al., 2015; Asgher et al., 2016). Although, only few reports explored the link between them under heat stress, action of NO as a downstream component in hormone-mediated pathways appears to be the common factor in thermotolerance (Mioto et al., 2014; Asgher et al., 2016). A possible role for NO as a downstream component in the auxin signaling pathway has been proposed in a series of plant growth and stress responses (Pagnussat et al., 2002; Hu et al., 2005; Chen et al., 2010; Kolbert et al., 2012; Freschi, 2013; Asgher et al., 2016). In addition to the impact of auxin on NO production, NO has been shown to influence auxin metabolism, transport and signaling (Fernández-Marcos et al., 2011; Terrile et al., 2012). Although, literature supports an intricate interconnection between these signaling pathways in various plant processes, their link has not been established under heat stress. However, in heat-stressed *N. tabacum* guard cell protoplasts, reduced NO levels lead to the suppression of auxin signaling suggesting NO as the central determinant of auxin-mediated cell fate (Beard et al., 2012). Further treatment with NOS-inhibitor L-N(G)-nitroarginine, photocopied the effects of heat stress in these protoplasts and seedlings by limiting cell division, expansion and dedifferentiation, and repressing the auxin-responsive promoter eventually resulting in reduced lateral root elongation, petiole length, and leaf expansion (Beard et al., 2012). This heat induced reduction of NO levels lead to the reduction of auxin signaling, that subsequently reduced ethylene production making most of cells quiescent. Thus far, a general antagonism has been observed between NO and ethylene for most of the physiological processes (Manjunatha et al., 2012; Melo et al., 2016) besides the indication of synergistic effects (Gniazdowska et al., 2007; Arc et al., 2013). In agreement with the reported antagonism of ethylene and NO, it is speculated that loss of root hair formation in *N. glauca* under heat treatment might be due to the scavenging effect of heat induced ethylene on NO (Beard et al., 2012). Numerous studies have implicated the role of ethylene in thermo-inhibition of seed germination (Arc et al., 2013). Induction of thermo-dormancy is correlated with reduced ethylene levels and ethylene biosynthetic gene expression, while exogenous ethylene could overcome the thermo-dormancy in *C. arietinum* and *A. thaliana* (Arc et al., 2013). One can attribute the observed inhibition of seed germination due to the increased NO levels as an indirect effect of the reduced ethylene levels under heat stress, a notion supported by increased seed germination upon NO scavenging. NO can modulate ethylene biosynthesis by reversibly inhibition of methionine adenosyltransferase through *S*-nitrosylation, leading to the reduction of the *S*-Adenosyl methionine pool or by stimulation of 1-aminocyclopropane 1-carboxylic acid-malonyltransferase activity thus diverting *S*-Adenosyl methionine pool toward the polyamine pathway (Lindermayr et al., 2006; Arc et al., 2013). Polyamines also have a significant role in heat tolerance in plants as evident by the altered heat sensitivity of transgenic plants overexpressing *spermine synthase* and a spermine deficient mutant plant of *A. thaliana* (Sagor et al., 2013). These results revealed the positive correlation between higher spermine content and thermotolerance. The reported functional overlap between polyamines and NO under other stress conditions and their co-occurrence in heat stress responses represents an rich scope for further interaction studies under heat stress (Tun et al., 2006; Fan et al., 2013; Nahar et al., 2016).

In addition, hydrogen sulfide (H_2_S)donor was also shown to improve heat tolerance in *N. tabacum* suspension culture cells (Li et al., 2015; Li and Jin, 2016) by enhancing calcium and proline levels NO has been suggested to act upstream to H_2_S in the acquisition of heat tolerance in *Z. mays* seedlings (Li Z. G. et al., 2013). SNP promoted the accumulation of H_2_S by stimulating the activity of L-cysteine disulfhydrase, that has resulted in enhanced heat survival percentage of *Z. mays* seedlings. While SNP-induced heat tolerance was augmented by the application of H_2_S donors such as sodiumhydrosulphideor GYY4137, its inhibitors/scavengers like DL-propargylglycine, aminooxyacetic acid, potassium pyruvate, hydroxylamine, and hypotaurine diminish heat tolerance (Li Z. G. et al., 2013). Sodiumhydrosulphide-pretreatment in *Fragaria ananassa* plants preserved photosynthesis by reducing MDA, H_2_O_2_ content and increasing the gene expression of antioxidants without any difference in NO levels under heat stress (Christou et al., 2014), thereby suggesting the operation of downstream to NO.

In another study, pretreatment with salicylic acid inhibited ethylene synthesis and increased nitrate reductase and γ-glutamyl kinase in *T. aestivum* leaves under heat stress. Although, not supported yet by experimental evidence, the observed reduction in ethylene levels and increased NR activity drives us to suggest the probable involvement of NO in this signaling cascade (Khan et al., 2013a,b; Khan M. I. R. et al., 2015). Stomatal closure is the other response where a possible downstream operation of NO and H_2_O_2_ has been suggested (Khokon et al., 2011; Zandalinas et al., 2016). Notably, the details of the actual mechanisms of NO and salicylic acid interaction in heat stress mitigation is not fully known. Heat induced stomatal closure in the leaves of *A. thaliana* has been observed due to enhanced NO levels triggered by the heat-induced H_2_O_2_, jasmonic acid, and repression of salicylic acid. Interestingly, NO-induced stomatal closure was also observed in ABA signaling mutant (*abi1-1)* suggesting an ABA-independent signaling triggered by NO (Zandalinas et al., 2016). Interestingly, in heat-stressed *P. communis* calli, inhibition of ABA synthesis by fluridone while showed no influence on the protective effect of exogenous NO, inhibition of NO accumulation by cPTIO and nito-L-arginine blocked the protective effect of exogenous ABA. Exogenous ABA further increased the heat induced NOS activity and NO release (Song et al., 2008) suggesting the role of NO as a key downstream molecule in ABA-dependent and -independent heat stress mitigation.

A broad spectrum of environmental stresses including heat stress trigger the accumulation of endogenous H_2_O_2_ (Lopez-Delgado et al., 1998). Exogenous H_2_O_2_ also enhanced thermotolerance in *Agrostis stolonifera* and *S. lycopersicum* microplants (Lopez-Delgado et al., 1998; Larkindale and Huang, 2005). Both NO and H_2_O_2_ operate in synergistic as well as antagonistic manner to serve as stress signals in plants (Neill et al., 2002) where their synthesis is synchronous during stress responses. Crosstalk between NO, H_2_S, and H_2_O_2_ has been reported in acquisition of heat tolerance in *Z. mays* seedlings (Li Z. G. et al., 2013). Nevertheless, feedback inhibition of H_2_O_2_ through the stimulation of antioxidant enzymes has conferred thermotolerance in plants (Wu et al., 2015). For example, SNP presoaking of *P. radiatus* leaf discs reduced subsequent production of H_2_O_2_ (Yang et al., 2006; Hasanuzzaman et al., 2013). While, increasing endogenous NO levels improved the heat tolerance in H_2_O_2_-deficient NADPH-oxidase mutants of *A. thaliana, atrbohB, atrbohD*, and *atrbohB/D*, enhanced endogenous H_2_O_2_ levels did not affect the NO-deficient *noa1* mutant, designating NO as a downstream factor in H_2_O_2_ signaling. Further, overexpression of *AtNIA2* or *AtNOA1* also restored stress acquisition in *atrbohB/D* mutant further confirming the relationship between NO and H_2_O_2_ in heat stress tolerance (Wang et al., 2014). In another study, pretreatment of *Z. mays* seedlings with H_2_O_2_ rapidly induced endogenous H_2_O_2_, NO, and H_2_S accumulation under heat stress, and this accumulation was reversed by H_2_O_2_ scavenger dimethylthiourea and NO scavenger cPTIO, indicating that H_2_O_2_ induced heat stress tolerance was involved in the crosstalk between downstream components NO and H_2_S (Li Z. G. et al., 2013).

Plant heat tolerance not just include crosstalk between H_2_O_2_, NO, and H_2_S, but also involve activation of Ca^2+^ channels, and Ca^2+^-CaM-dependent protein phosphatase along with other factors to induce DNA-binding activity of the heat shock factors and subsequent accumulation of HSPs (Wang et al., 2014). NO acted upstream of *AtCaM3* in *A. thaliana*, inducing the DNA-binding activity of heat stress transcription factors and the abundance of HSP18.2 (Xuan et al., 2010) suggesting that NO acts in a systematic signaling pathway recruiting other signaling molecules timely at the right location in order to achieve heat stress tolerance. So far, while the crosstalk between these signaling molecules and events has been well studied in drought stress, not much information is available under heat stress. Hence, further investigations in this direction are imperative to develop a complete network of signal transduction in response to heat stress.

## Hypothetical mechanism of NO induced heat tolerance in plants

Plants adapt to high temperatures by a series of events beginning with the stress perception and end at the expression of target genes. Plants perceive heat stress by perturbation to cellular homeostasis that triggers stress signaling, probably by transducers such as NO, H_2_O_2_, Ca^2+^, and other stress hormones like ABA. These signaling molecules in turn induce the synthesis of selected protein kinases that stimulate the downstream gene expression. The altered gene expression often leads to cascade of events including changes in plant metabolism, synthesis of antioxidants, accumulation of osmoprotectants, synthesis of HSP, and enhanced survival under heat stress (Farooq et al., 2011).

Previous works have provided evidence for the involvement of NO at various levels of thermo-tolerance (Table 1). Various signaling components/molecules, transcription regulation elements, candidate genes, and proteins are involved in NO-mediated stress response, including the stress induced morphological, physiological, biochemical, and molecular changes. Reports suggest that NO acts downstream of H_2_O_2_ in the heat stress signaling in *A. thaliana* seedlings (Wang et al., 2014). However, as mentioned earlier, a feedback inhibition of H_2_O_2_ levels by the action of antioxidant enzymes by NO is known to inhibit heat shock factor activity and HSP production. Network of H_2_O_2_, NO, Ca^2+^ channels, and Ca^2+^-CaM dependent protein phosphatase was also reported to mediate heat shock factor activity and HSP accumulation in heat stress tolerance (Wang et al., 2014). Exogenous NO donors such as SNP might activate NADPH oxidases, subsequently enhancing the production of Ca^2+^ and ROS. H_2_S-induced expression of NiR and mitochondrial NAD(P)H dehydrogenases has been reported in *Citrus aurantinum* roots. However, the NO production by this pathway still needs to be proven under heat stress (Gupta et al., 2011). A recent study in *S. lycopersicum* seedlings subjected to chilling stress indicated that an exogenous treatment with NO enhanced putrescine and spermidine levels and application of spermine and spermidine stimulated NO production in a H_2_O_2_-dependent manner through both NR and NOS-like pathways. Although, NO has short half-life, its functionality can be extended in the form of SNOs which can release NO in a controlled manner. Heat stress induced a significant increase in the total SNOs content in *C. morifolium* while decreasing the NO and GSNOR levels (Chaki et al., 2011). Moreover, the functional existence of NO-mediated PTMs add more complexity and interaction nodes in heat stress signaling. The experimental proofs so far obtained under heat stress, and the predicted possible interactions with other signaling molecules strongly support the central role played by the NO in plant heat stress signaling. However, an intensive future research is required to have complete picture that mechanistically explain “how and where” NO fits in the heat stress signaling.

## Future perspectives

Elevated temperatures have greater and negative impact on plants. Despite of the methodological difficulties, recent literature clearly indicates the various roles played by NO in mediating heat stress responses in plants (Figure 2). Owing to its concentration dependent cytoprotective and toxic role, it is essential to have controlled release of NO in plant cells especially under stress conditions. Some recent reviews suggest encapsulation of NO donors in nanomaterials for an improved efficiency and controlled release over direct exogenous applications (Seabra et al., 2014; Savvides et al., 2016). This kind of target site application of nanoparticles also offer high potential due to reduced cost of application, precise dosing, decreased decomposition due to environmental factors, reduced runoff of unutilized excess chemicals into environment. Further exploration of smart technological approaches and deployment of these nanoparticles can allow effective release of NO minimizing side effects. While pretreatment with NO donors has shown promise in increasing heat tolerance in plants, it is critical to understand the methods of stress treatment and NO loading, and the tissue type used to explain the contradictions in reported data. Since the sensitivity to heat varies from species to species, mere extrapolation to other species is not practical to achieve heat stress tolerance at field level.

Several studies have used NO at different plant growth and developmental stages to understand its role in plant heat stress tolerance (Table 4). Although, these efforts do add to our understanding, we advocate the importance of developmental stage-specific studies of NO-mediated thermotolerance, especially in crop plants because many legumes and cereals show high heat sensitivity during reproductive development due to restricted water and nutrient transport under stress (Young et al., 2004; Devasirvatham et al., 2012). Hence, it is essential to put more efforts in NO-mediated stress tolerance research in crop plants to validate its implications at the field level by moving from lab to field experiments. This would allow stress ameliorating effects of NO to be translated to yield benefit in the context of anticipated climate change. Indeed, the most attributed NO function in heat tolerance is the stimulation of antioxidants (Bavita et al., 2012; Hasanuzzaman et al., 2012), however, merely increasing survival without enhanced biomass and yield again might not be practical for crop improvement.

**Table 4:** Different types of plant tissues used for nitric oxide treatment in plant heat stress studies.

**Plant tissue**	**NO donor**	**Plant species**	**Plant process studied**	**References**
Seeds	SNP	*Arabidopsis thaliana, Lactuca Sativa*	Seed germination, de-etiolation, hypocotyl and intermodal elongation	Beligni and Lamattina, 2000; Hossain et al., 2010
Seedlings	SNP DETA/NO	*Triticum aestivum, Arabidopsis thaliana, Oryza sativa, Zea mays*	Lipid peroxidation, oxidative damage and NO signaling	Lee et al., 2008; Xuan et al., 2010; Bavita et al., 2012; Hasanuzzaman et al., 2012; Li Z. G. et al., 2013; Song et al., 2013
Leaves	DETA/NO& SNP	*Phaseolus radiatus, Nicotiana tabacum, Arabidopsis thaliana, Festuca arundinacea, Chrysanthemun morifolium*	Photosynthesis, Membrane damage, osmolyte accumulation and anti-oxidant defense	Gould et al., 2003; Yang et al., 2006; Lee et al., 2008; Yang W. et al., 2011; Chen et al., 2013
Roots	SNP	*Gingiber officinale*	Membrane damage and anti-oxidant defense	Li X. et al., 2013
Mature plants	SNP	*Fragaria ananassa, Oryza sativa*	Membrane damage, anti-oxidant defense, HSP accumulation	Uchida et al., 2002; Christou et al., 2014
Callus	SNP & SNAP	*Phragmites communis, Triticum aestivum*	NO acts as signaling in conferring heat tolerance and reduces oxidative damage in plants	Song et al., 2006; El-Beltagi et al., 2016
Cell suspension	SNAP	*Nicotiana tabacum*	Heat stress at 55°C resulted in rapid surge in NO while no detectable increase in NO was observed at 35°C	Locato et al., 2008

A major knowledge gap in NO-heat stress studies is the lack of in depth molecular understanding. So far, most of the reports on the exploration of beneficial potential of NO under heat stress have been based on the physiological and biochemical studies. Hence, it is crucial to develop high-throughput genomic, proteomic, and metabolomics data and its integration at the systems level to understand the impact of heat induced accumulation of NO in plant tissues. Not only it is worth for establishing correlations between NO content and the gene/protein expression, with special emphasis on its subcellular location; but also the role of NO-mediated PTMs in heat stress mitigation. It is also essential to address the change in scenario of heat stress signaling mechanism and the NO-interacting partners when plants simultaneously challenged by other stress conditions in the field. Moreover, it is important to develop metabolite profiles of various durations might potentially provide deep insights into the functional intermediary or signal transduction components involved in exogenous NO-mediated response of plants to heat stress and various donors (León et al., 2016). Futuristic tools based on the emerging genome editing technologies also have tremendous potential in uncovering the NO signaling components and identification of the key genes involved in the NO-mediated thermotolerance. Since the accumulating data is establishing NO as a key role player in heat acclimation, clear insights into NO signaling networks underpinning the plant heat stress responses using newer tools and technologies might offer novel opportunities for rational crop design to ameliorate heat stress impacts.

## Author contributions

SP conceived the idea and wrote the first draft. SP, SA, PB and KS contributed to the writing and refining of the manuscript.

### Conflict of interest statement

The authors declare that the research was conducted in the absence of any commercial or financial relationships that could be construed as a potential conflict of interest.
